# Double UP: A Dual Color, Internally Controlled Platform for *in utero* Knockdown or Overexpression

**DOI:** 10.3389/fnmol.2020.00082

**Published:** 2020-05-20

**Authors:** Russell J. Taylor, Justin Carrington, Leah R. Gerlach, Kendra L. Taylor, Karl E. Richters, Erik W. Dent

**Affiliations:** ^1^Neuroscience Training Program, University of Wisconsin-Madison, Madison, WI, United States; ^2^Department of Neuroscience, University of Wisconsin-Madison, Madison, WI, United States

**Keywords:** *in utero* electroporation, migration, plasmid–gene delivery, development neuroscience, overexpression and knockdown

## Abstract

*In utero* electroporation (IUE) is a powerful tool for testing the role of genes in neuronal migration and function, but this technique suffers from high degrees of variability. Such variability can result from inconsistent surgery, developmental gradients along both rostral-caudal and medial-lateral axes, differences within littermates and from one litter to another. Comparisons between control and experimental electroporations rely on section matching, which is inherently subjective. These sources of variability are cumulative, leading to difficult to interpret data and an increased risk of both false positives and false negatives. To address these limitations, we developed two tools: (1) a new plasmid, termed Double UP, which combines LoxP-flanked reporters and limiting Cre dosages to generate internal controls, and (2) an automated program for unbiased and precise quantification of migration. In concert, these tools allow for more rigorous and objective experiments, while decreasing the mice, time, and reagents required to complete studies.

## Introduction

The method of *in utero* electroporation (IUE) (Saito and Nakatsuji, 2001; Tabata and Nakajima, 2001) has contributed greatly to our understanding of neuronal differentiation and migration in the central nervous system. Indeed, it is the *de facto* technique to determine how neuronal function and migration are disrupted in the central nervous system after genetic manipulation or in disease models.

However, this technique relies on “section matching” to generate controls, an approach which suffers from a high degree of variability due to a multitude of inherent challenges, including inconsistent surgeries, developmental differences within and between litters, gradients of maturation within multiple axes in the developing brain (Bayer and Altman, 1991), and inconsistent section matching between control and experimental conditions. These sources of variability are cumulative, leading to difficult to interpret data and an increased risk of both false positives and false negatives.

To overcome these limitations inherent in IUE, we have developed a novel plasmid containing LoxP-flanked reporters termed Double UP, designed to generate optimal internal controls. By titrating the amount of Cre transfected with Double UP, we are able to label approximately half of the neurons with a green reporter, which serves as a control, and the other half of neurons with both a red reporter and an experimental manipulation, either protein overexpression or shRNA-mediated knockdown. Thus, both green (control) and red (experimental) neurons are present throughout the electroporated area. When the electroporated area of cortex is sectioned and imaged, green and red cells can be quantified in a single slice, greatly reducing the dependency on section matching.

## Results

### Introducing an Internal Control to *in utero* Electroporation

**FIGURE 1:**
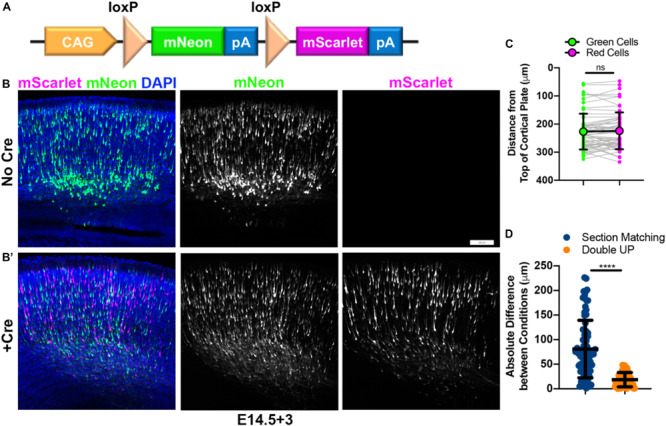
Construction and characterization of Double UP. Double UP generates equivalent populations of red and green neurons within an individual slice. **(A)** Schematic of Double UP construct. Representative IUE of 2 μg/μL Double UP without Cre **(B)** and with 15 ng/μL pCAG-Cre **(B’)**. Embryos were electroporated at E14.5 and allowed 3 days to mature (E14.5+3). Scale bar, 100 μm. **(C)** Comparison of migration of mNeon-Green- and mScarlet-positive cells, E14.5+3. Each dot represents the mean distance of all cells within one cortical slice to the top of the cortical plate. Connected dots indicate measures from the same slice (*n* = 61 slices). Large dots and black line indicate mean and SD of all 61 slices. No significant difference was detected between green and red populations (two-way ANOVA). Full data shown in Supplementary Figure S1. **(D)** Comparison of reliability of controls between section matching and Double UP. 76 comparisons were made for section matching and 61 comparisons were made for Double UP. Only sections that had a perfect match in another brain were included for either analysis. ****p < 0.0001, Kolmogorov–Smirnov *t*-test, two-tailed. Lines indicate mean and SD. See also Supplementary Figure S1.

To address and mitigate the variabilities inherent to IUE, as well as the additional confounds of studies utilizing mixed genetic backgrounds, we have developed a dual-fluorescent plasmid, designed to generate an internal control. This plasmid, termed “Double UP,” uses the strong ubiquitous CAG (*C*MV enhancer/beta-*A*ctin promoter and rabbit *G*lobin PolyA tail) promoter (Kootstra and Verma, 2003). The CAG promoter is followed by a LoxP-flanked cassette (Kilby et al., 1993) consisting of the green fluorescent protein mNeon-Green (Shaner et al., 2013), a stop codon and the rabbit-globin PolyA tail (Lanoix and Acheson, 1988). Following the second LoxP site is the red fluorescent protein mScarlet (Bindels et al., 2017), with an identical stop codon and rabbit-globin PolyA tail (Figure 1A). In the absence of Cre, cells containing this plasmid produce mNeon-Green (Figure 1B). However, if Cre is present in a cell, the mNeon-Green is excised, and replaced in the exact same locus by mScarlet. Co-injection with a limiting dose of pCAG-Cre (Matsuda and Cepko, 2007) plasmid allows rough control of the ratio of green cells to red cells. For cortical IUE, we titrated the amounts of Cre plasmid from 1 ng/μL to 1 μg/μL, and found that 15 ng/μL of Cre plasmid results in roughly half green cells and half red cells (Figure 1B’) and have used that concentration throughout the manuscript, unless otherwise noted. Greater than 200 embryos have been transfected with pCag-Cre at 15 ng/μL, the large majority have had roughly equivalent numbers of green and red cells, with fewer than 10% having had at least twice as many cells of one color as the other. This Cre concentration will likely vary depending on usage, based on region targeted, timing of electroporation, amount of DNA being injected, and the exact pulse sequence used during IUE. The same promoter (CAG) is used in both Double UP and Cre, so no preferential cell selection should occur, a hypothesis rigorously tested below.

### Automated Quantification of Data

**FIGURE 2:**
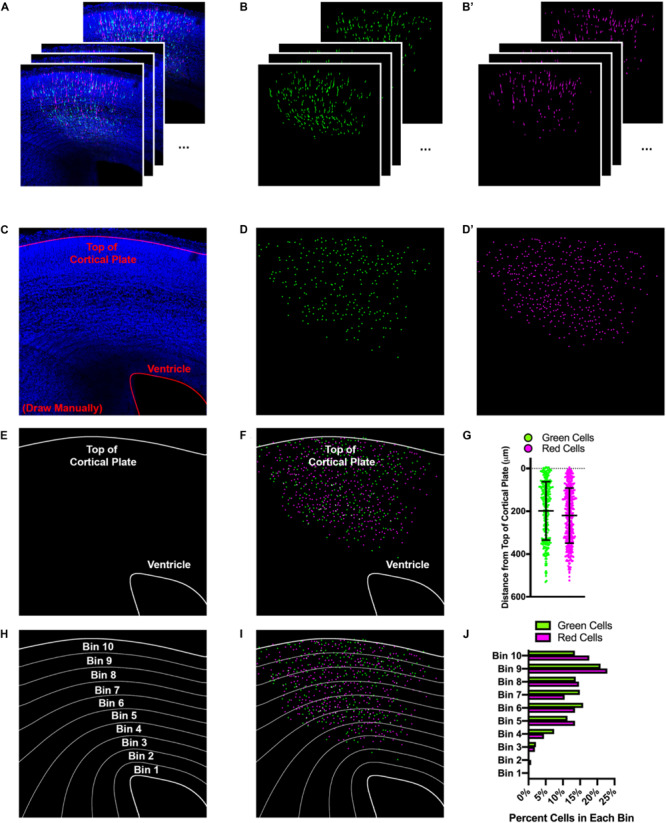
Automated tracking of cellular location with TRON program. Example of data analysis using the TRON program. **(A)** Example input. 12, three-color confocal slices from a 100 μm coronal brain slice from an E14.5+3 embryo after IUE. **(B)** Green and **(B’)** red colors, processed independently and automatically via: unsharp mask, gaussian blur, conversion to 8-bit, automatic threshold, erode, watershed. **(C)** Manual drawing of regions of interest (ROIs—red lines at top of cortical plate and ventricle). This is the only manual step in the process. **(D,D’)** Output of modified 3D Object Counter plugin from Fiji. Center of mass of each cell is identified and mapped; *X*, *Y* location and brightness of original is obtained and recorded. **(E)** Tracings of the top of the cortical plate and ventricle (ROIs). **(F)** Location of each cell mapped onto the ROIs. Distance from the center of each cell to the nearest point on each ROI is measured and recorded. **(G)** Graphical representation of distance each cell is from top of the cortical plate. Cells located above the top of the cortical plate are treated as having a “negative” distance. Lines indicate mean and SD. **(H)** Distance between ROIs can be split into bins, calculated to reflect the ROI to which they are closest. **(I)** Location of each cell mapped onto the bins. **(J)** Graphical representation of the data split into 10 equal-sized bins, each bar shows percentage of cells of that color in the respective bin.

It became clear as we were developing Double UP that having a more precise and quantitative way of measuring differences in migration between control and experimental conditions would provide a more robust measure of migratory changes after protein overexpression or knockdown. Prior approaches to IUE analysis typically relied on linear bins to separate different regions of cortex, so that data could be easily combined and compared across brains. With the ability to generate control and experimental cells within the same hemisphere, we set out to develop an automated method of quantification, designed to give exact positional information for each cell. To accomplish this, we developed a program which allows for automated quantification of distance from the nearest point on any user-defined region of interest (ROI), as well as location and brightness. This Java-based program, entitled “TRacking Overlapping Neurons” (TRON) utilizes simple processing steps, followed by a modified version of the Fiji plugin 3D Object Counter (Bolte and Cordelieres, 2006). TRON identifies the center of mass of every fluorescently-labeled neuronal cell body and calculates the distance to any user defined ROI (Figures 2A–G). This approach eliminates the need for linear binning and manual counting, avoiding both data compression and user bias. For comparison with historical data, TRON does have the option to present data in automatically generated bins, with curvature that conforms to the actual curvature of cortical sections (Figures 2H–J). This process is highly automated, reducing the prevalence of bias, while simultaneously simplifying labor intensive data analysis. The only manual steps in this analysis program are demarcating regions of interest and optional “quality control” of neuronal location. The individual processing steps are available in Online Methods. The TRON program, a sample image and associated instructions are downloadable at https://go.wisc.edu/tron. We implement this program throughout the rest of the manuscript.

### Confirming That Green and Red Neurons Behave Similarly

To test the validity of Double UP, we needed to determine if the green and red fluorophores were either differentially affecting neuronal migration in the absence of overexpression/knockdown, or were labeling dissimilar populations of cells. If both mNeon and mScarlet are inert and labeling unbiased populations of cells, the green and red population of neurons should migrate similarly. To test this assumption, we performed surgery and imaged every section containing fluorescent cells from four embryos (*n* = 61 sections, four embryos, from two pregnant mice). Sections were collected and mounted sequentially and aligned across brains according to the first section which contained an uninterrupted corpus callosum (CC). This matching scheme corresponded well with other common landmarks. Section matching refers to the comparison of all fluorescent cells (mNeon or mScarlet) between matched sections from two different brains. “Double UP” refers to the comparison between both mNeon and mScarlet cells within one section of one brain. Implementing the TRON program, referred to above, we were able to determine that mNeon-Green and mScarlet-labeled neurons migrate in statistically similar fashions in embryonic brain (Figure 1C and full data set in Supplementary Figure S1). We decided to analyze migration as the distance from the top of the cortical plate, defined by the end of the DAPI labeled cell dense region. At the timepoints we examined, this is a measure of distance remaining to travel, as neurons will continue to migrate until they reach the top of the cortical plate. We also analyzed the distance of neurons from the ventricular surface, but ultimately decided to use the distance from the top of the cortical plate, to better isolate radial migration as opposed to the combination of radial and tangential migration. In control situations, measuring distance from the ventricular surface does not result in significantly different migration patterns between green and red cells (data not shown). Any cells located beyond an ROI were included in analysis and resulted in a negative value. Binned data were also collected and analyzed, but not used because binning inherently requires compression of data and therefore loss of information. Because all data from Double UP consist of matching sets of green and red cells, more powerful statistical tests (two-way ANOVA) can be used to determine if red and green neurons are behaving differently. Together, these data indicate that Double UP is appropriately generating internal controls, and allows for its use to compare control and experimental conditions in single brain sections.

### Comparing Traditional IUE and Double UP

One confounding variable in traditional IUE is the requirement of using matched sections in control and experimental brains. It is well established that embryonic cortical development proceeds along both rostral-caudal and medial-lateral gradients (Bayer and Altman, 1991). However, the degree to which these gradients cause variabilities to IUE has not been well documented. To determine the validity of section matching in light of these gradients, we re-examined the data from Figure 1C. Neuronal migration data were compared in two ways. First, to perform section matching, all sections were aligned using the first slice within each brain to contain an uninterrupted CC as a landmark. After alignment, all fluorescent cells from each section were compared with all fluorescent cells from matching sections from the other brains (see large box spanning in Supplementary Figures S1a,b as an example). We believe this is more rigorous than what is typically considered section matching; however, publications that implement IUE often do not typically report the methodology used to section match, or even if section matching was implemented. Second, to compare with Double UP, each section that had at least one matching section was then also analyzed (see small box in Supplementary Figure 1b as an example). The mean migration distance of green cells and red cells was compared within each section (Figure 1D). Between matched sections, we found an absolute variance of 80.8 ± 6.7 μm (SEM). Utilizing Double UP, we found an absolute variance of 18.6 ± 1.9 μm. Within Double UP, there was negligible bias toward either color migrating further, with green cells migrating 2.6 ± 3.0 μm further than red cells. If even precise section matching has high degrees of variability, it calls into question the practice of using matching sections from separate electroporations as a methodology to compare control and experimental conditions.

### Testing for Leakiness

**FIGURE 3:**
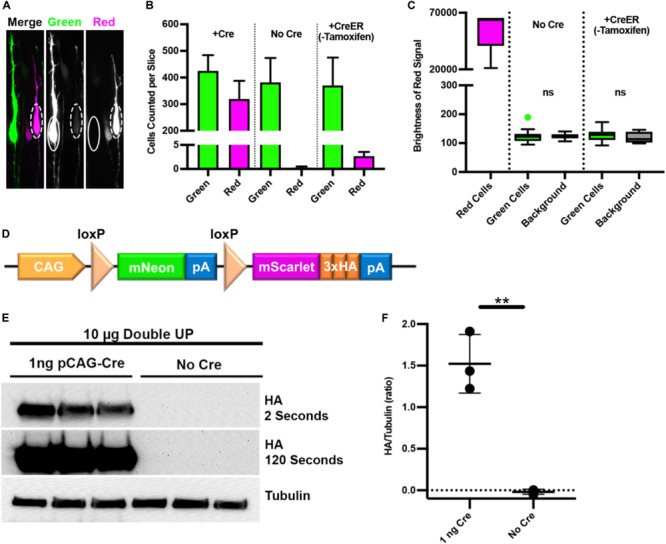
Double UP is not “leaky.” Three tests to determine if green cells express any red protein or associated manipulation. **(A)** High power image of two adjacent neurons, one green and one red, taken with a 63x/1.4NA oil objective. In the green neuron (solid oval), no signal is visible in the red channel. In the red neuron (dashed oval), a faint signal is visible in the green channel. **(B)** Number of green and red cells of greater than 1000 units of brightness per slice in Double UP +Cre (three slices, mean = 425 green/320 red cells), Double UP No Cre (four slices, mean = 382 green/0.25 red cells), and Double UP +ERT2-Cre-ERT2 (three slices, mean = 370 green/three red cells). Error bars indicate SEM. All images acquired with identical settings. **(C)** Quantification of the brightness in the red channel of red neurons from 90 neurons across three brains for: red neurons from Double UP +Cre, as well as green neurons from brains having received Double UP without Cre and Double UP +CreER. Dot above box and whiskers in “Green Cells” column with No Cre indicate an outlier. All other points are contained within the whiskers (Tukey). Boxes are 25/75%, median is also shown. **(D)** Schematic of Double UP-HA. **(E)** Western blots of protein from CAD cells transfected with 10 μg of Double UP and either 1 ng/μL pCAG-Cre or no Cre. Quantification done on HA exposed for 2 s, 120 s exposure only shown for reference. **(F)** Amount of HA protein detected in each condition. The average HA signal in three experiments was slightly less than background. **p = 0.0017, unpaired two-tailed *t*-test of three experimental replicates. Lines indicate mean and SD.

The intent of Double UP is to introduce overexpression or knockdown in combination with the red fluorophore to distinguish if and how the red experimental cells differ from the green control cells. It was expected that some red cells would be dimly green, and this was observed (dashed circles, Figure 3A). Red cells that were dimly green were expected because both Double UP and Cre share the same CAG promoter and therefore mNeon will be transcribed simultaneously with Cre prior to Cre-mediated recombination of Double UP. Examining seven brains electroporated with Double UP and pCAG-Cre, 1.0% of red cells had a green signal bright enough to be identified as both red and green cells by the TRON program. We have introduced a test in TRON to detect and disregard double positive cells, which was implemented for all studies other than those testing for leakiness. We believe double positive cells to be a result of rare instances when either Cre plasmids/protein transcribe or translate slowly, or when one or more copies of Double UP are slow to recombine for reasons that are unclear. Because it is expected that the first fluorophore will always be expressed at least briefly, any overexpression or knockdown is always associated with the second fluorophore.

It is acceptable, and likely unavoidable, for red (Cre-positive) cells to express mNeon. However, for the success of Double UP, it is vitally important that green (Cre-negative) cells do not express any mScarlet (solid oval, Figure 3A) or any associated overexpression or knockdown; a potential problem we are defining as “leakiness.” If Double UP was leaky, putative control cells would have either overexpressed protein or shRNA, and therefore be inappropriate controls. The design of Double UP also allows for delayed activation of both the red fluorescent protein and overexpression or knockdown, by transfecting limiting doses of CreER and timed administration of tamoxifen. We therefore also tested the system for leakiness using pCAG-ERT2-Cre-ERT2 (Matsuda and Cepko, 2007) (pCAG-CreER), without administration of tamoxifen. To test for leakiness, we undertook multiple tests. First, IUE was performed with Double UP (2 μg/μL) and either no Cre, pCAG-CreER (15 ng/μL), or pCAG-Cre (15 ng/μL). Sections from the pCAG-Cre brains were used to establish settings such that a few pixels were saturated, and then unchanged between conditions. Rather than using automatic thresholding for the TRON program, images for this set of experiments were artificially thresholded at 1000 gray values, out of 65,535 possible. This gray value was an artificially low threshold, designed to detect even very dim cells. In the absence of Cre, there was a single red cell slightly above this threshold across four sections from four different brains (Figure 3B, column 4). IUE of Double UP and pCAG-CreER resulted in three to four red cells in each section (compared with several hundred green cells) (Figure 3B, column 6), and IUE of Double UP and pCAG-Cre resulted in roughly equal populations of green and red cells (Figure 3B, columns 1 and 2). These results demonstrate that in Cre negative cells, there is virtually no recombination of Double UP. It was not a surprise that Double UP with CreER (without tamoxifen) was more leaky than Double UP without Cre, as CreER has been previously shown to have a low level of activity in the absence of exogenous tamoxifen/estrogen (Matsuda and Cepko, 2007). This is a known but acceptable tradeoff for the ability to have temporal control of activation.

Absence of visibly red cells in the no-Cre condition suggests that the loxP sites within Double UP are not recombining. However, this result does not establish whether green cells had below visible expression of mScarlet, or any associated manipulation. To test this, we needed to quantify the intensity of the red signal in individual green cells. Using the same images that were used in the previous leakiness test, fluorescent intensity measures were collected for 90 green cells in no-Cre and CreER conditions, and from 90 red cells in a +Cre condition, from three different brains per condition. In both no-Cre and CreER conditions, the brightness in the red channel was unchanged between green cells and background levels (Figure 3C), also indicating that Double UP is not leaky.

Finally, we performed Western blotting to detect any protein associated with mScarlet. To accomplish this, we used Double UP 3x-HA, in which 3x-HA was fused with the mScarlet fluorophore (Figure 3D). Western blotting analyses, performed in a mouse catecholaminergic cell line (CAD cells), demonstrated that in the presence of very little Cre plasmid (1 ng), HA protein is readily detectable, but in the absence of Cre, there is absolutely no detectable HA protein (Figures 3E,F). Together, these data suggest that in the absence of Cre, no mScarlet or associated protein is produced, and therefore that Double UP is not leaky.

### Replicating Previous Findings: Overexpression

**FIGURE 4:**
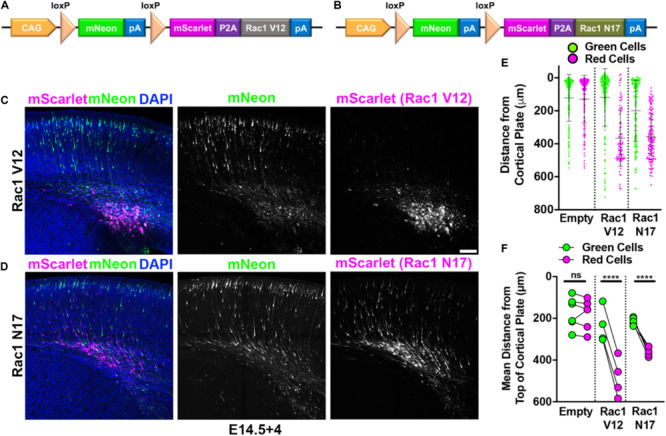
Double UP provides overexpression with an internal control. Robust replication of previously discovered migration defects utilizing Double UP. **(A)** Schematic of Double UP Rac1-V12. **(B)** Schematic of Double UP Rac1-N17. **(C)** Representative image of Double UP Rac1-V12, E14.5+4. Scale bar, 100 μm. **(D)** Representative image of Double UP Rac1-N17 E14.5+4. **(E)** Dot plot of three representative slices from Double UP, Double UP Rac1-V12, and Double UP Rac1-N17. Each dot represents distance from the top of the cortical plate to the center of mass for each neuron. **(F)** Dot plot of Double UP, Double UP Rac1-V12, and Double UP Rac1-N17 (*n* = 6, 4, and 5 slices, respectively, each from a different brain). Each dot represents mean distance from the top of the cortical plate to the distance of all cortical neurons in a slice. Connected dots indicate measurements were made in the same brain. ns = not significant, ****p < 0.0001, two-way ANOVA. Error bars not shown for clarity. See also Supplementary Figure S2.

In the absence of protein overexpression, red and green neurons migrate equivalently (Figure 1C). This allows for including overexpression of a protein of interest with the red fluorophore and examining the effects of this overexpression relative to the control green neurons. Thus, we set out to determine if using Double UP recapitulated previously published findings from multiple groups. Interestingly, both constitutively active Rac1 (Rac1-V12) and dominant negative Rac1 (Rac1-N17) have been shown previously to inhibit neuronal migration (Kawauchi et al., 2003; Konno et al., 2005). Rac1-V12 and Rac1-N17 were separately cloned downstream of mScarlet, following a P2A peptide (Figures 4A,B). The P2A causes a ribosomal skip during translation, so that equimolar ratios of both mScarlet and either Rac1-V12 or Rac1-N17 are generated. Cells receiving both Double UP Rac1-V12 or Double UP Rac1-N17 and at least one Cre plasmid will now produce mScarlet and an untagged Rac1-V12 or Rac1-N17. Based on the leakiness experiments completed above, cells receiving only Double UP will produce only mNeon. IUE was performed at E14.5 and collected 4 days later (E14.5+4). Red neurons expressing either Double UP Rac1-V12 or Double UP Rac1-N17 fail to migrate, while green neurons migrate normally (Figures 4C–F and full data in Supplementary Figures S2a–c), indicating that Double UP replicates previous findings. Importantly, green neurons in each condition are not significantly different from green neurons in each other condition (one-way ANOVA, *p* = 0.33), further demonstrating that Double UP is not leaky, while also suggesting that Rac1-V12 and Rac1-N17 both act in a cell-autonomous manner.

### Replicating Previous Findings: Knockdown

The second common manipulation performed via IUE is knockdown, and there are already existing multiple Cre-dependent knockdown strategies (Coumoul et al., 2004; Ventura et al., 2004). After multiple rounds of design and experimentation, we determined that pSico PGK-Puro (Ventura et al., 2004) provided the strongest and most reliable Cre-dependent knockdown. This plasmid has also been demonstrated previously to absolutely require Cre to generate knockdown (Ventura et al., 2004).

**FIGURE 5:**
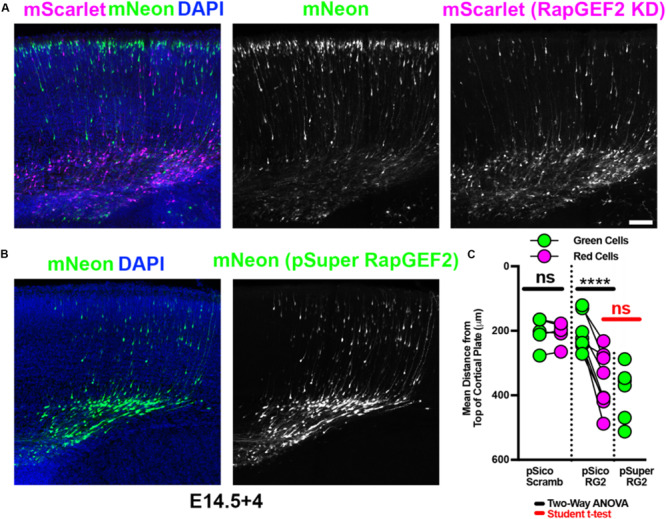
Double UP combined with pSico provides knockdown with an internal control. Robust replication of previously discovered migration defects, utilizing Double UP and pSico. **(A)** Representative image of IUE with pSico RapGEF2 (2 μg/μL), Double UP (1 μg/μL), and pCAG-Cre (15 ng/μL). Scale bar: 100 μm. **(B)** Representative image of IUE with pSuper RapGEF2 (2 μg/μL) and Double UP (1 μg/μL). **(C)** Dot plot of Double UP and either pSico Scrambled (pSico Scramb), pSico RapGEF2 (pSico RG2), and pSuper RapGEF2 (pSuper RG2) (*n* = 5, 8, and 5 slices, respectively, each from a different brain). Each dot represents mean distance from the top of the cortical plate to all cortical neurons in a slice. Connected dots indicate measurements were made in the same brain. ns = not significant, ^****^p < 0.0001. Comparisons within samples (black bars) were two-way ANOVA; comparison between pSuper RapGEF2 and the red cells of pSico RapGEF2 (red bars) were performed with a Student’s *t*-test. Error bars not shown for clarity. See also Supplementary Figure S3.

RapGEF2 knockdown has previously been shown to cause a decrease in migration (Ye et al., 2014). We were able to recapitulate this phenotype using pSico PGK-Puro RapGEF2, to the same extent seen with pSuper RapGEF2, utilizing identical shRNA sequences (Figures 5A–C and full data in Supplementary Figure S3). Importantly, we saw no significant difference between migration of green and red cells when a scrambled shRNA was used (Figure 5C and full data in Supplementary Figure S3a). These findings indicate that Double UP and pSico PGK-Puro can replicate previous findings, to the same extent as pSuper. As with overexpression, the green cells receiving pSico Scrambled were not significantly different than the green cells receiving pSico RapGEF2 (*p* = 0.8890, Student’s *t*-test). This suggests that pSico is not leaky, and that the effects of knocking down RapGEF2 are cell autonomous.

## Discussion

*In utero* electroporation has been a viable experimental technique for almost 20 years, with minimal alterations to the original protocols. However, the robustness of an experiment is dependent on the reliability of the controls used. Overreliance on section matching is a problem with traditional IUE, as variations between electroporations or between embryos can lead to significant shifts in migratory patterns. Section matching may also compromise other measures not tested here, including cell fate decisions, axonal targeting, or dendritic branching. Many publications utilizing IUE neither refer to section matching nor littermate controls, which potentially introduces new confounds, since available measures to reduce variability may not have been appropriately undertaken. The technique of IUE is challenging, requiring a dedicated teacher and patient researchers to master. Migration profiles can vary 80 μm on average and as much as 220 μm even between precisely matched sections (Figure 1D). It is for these reasons that implementing Double UP, with or without pSico, will quantitatively improve an already powerful technique.

The concept of a green/red plasmid dependent on Cre expression is not in itself novel. A green/red plasmid termed “Stoplight” was first introduced in 2001 (Yang and Hughes, 2001), and has been used in many lineage tracing studies. Double UP is essentially a “modernization” of Stoplight, using the same basic design, but with brighter and more stable fluorophores, and a stronger promoter. Other groups have used LoxP-based green/red plasmids as markers for labeling cells before or after Cre expression (D’Astolfo et al., 2015; Fernandez-Chacon et al., 2019). These techniques and approaches have been used for lineage tracing, or as reporters for Cre expression within floxed alleles. The novelty of Double UP resides in the simple but critical technique of using limiting dosages of non-selective Cre to pseudorandomly select cells to be either control or experimental, and thus generating internal controls.

Development and validation of Double UP was remarkably quick and efficient. In contrast, development and validation of a suitable Cre-dependent knockdown was slow and arduous. We attempted four different versions of plasmids which would constitutively express a scrambled shRNA/miRNA, to be replaced by a gene-of-interest targeting shRNA/miRNA following Cre recombination. All attempts knocked down gene expression *in vitro*; however, none were capable of reliably reproducing phenotypes *in utero*. Following these attempts, we began using pSico PGK-Puro (Ventura et al., 2004) as a Cre inducible shRNA. pSico does not constitutively express an shRNA or miRNA, and so does not produce ideal internal controls. However, as only pSico reliably replicated *in utero* phenotypes, we selected it despite this caveat. It is unclear why the other strategies failed, though it is possible that production of shRNA interfered with Cre recombination, or vice versa.

With traditional IUE, it is often unclear if an effect is cell-autonomous or cell non-autonomous. Untransfected cells are not fluorescently labeled, and in an extremely crowded cortex, it can be exceedingly difficult to determine the morphology of non-fluorescent cells. Groups have previously attempted to address these concerns by performing sequential IUE, first introducing control plasmids and after some time experimental plasmids or vice versa. The interval between these sequential electroporations has been as little as 15 min (Baek et al., 2015) to as long as 24 h (Zhang et al., 2012). However, in this context, it can be difficult to determine if cells have overlapping expression of control and experimental plasmids at short time windows, and at longer time windows, it is no longer possible to test for cell autonomy in neurons born at similar times. Use of Double UP provides a tool to address the question of cell autonomy. As leakiness has been rigorously tested in Double UP (Figure 3), researchers can say with confidence that a green cell does not contain any scarlet or associated manipulation. In this manuscript, we tested three different experimental conditions (Rac1-V12 overexpression, Rac1-N17 overexpression, and RapGEF2 knockdown). In each experimental condition, the migration pattern of green (control) cells was not significantly different than the migration pattern of green cells in control conditions (empty or scrambled RNAi), suggesting that all experimental manipulations were cell-autonomous effects. Thus, comparing green cells in control and experimental conditions could address questions of cell autonomy/non-autonomy for overexpression or knockdown of any protein of interest.

An additional means of decreasing protein expression is to utilize transgenic animals containing two floxed alleles of a gene of interest. IUE of Double UP and limiting dosages of Cre plasmids would label presumed knockout cells red, and wildtype cells green. However, there are concerns of incomplete recombination with low dosages of Cre and floxed alleles (Liu et al., 2013). Use of Double UP will not eliminate those concerns. Thus, to allow for combinatorial experiments utilizing transgenic mice and Double UP, we have generated and tested an additional plasmid (Double UP-FRT), which utilizes FLPe-FRT recombination (Akbudak and Srivastava, 2011) to achieve the same results as Double UP, while not interfering with Cre-based systems (data not shown). While this plasmid is not utilized in this manuscript, it has been deposited along with Double UP to Addgene.

*In utero* electroporation is a technique that has been used in a broad range of scientific studies, not limited to cortical neuron migration. Examples include studies focused on ganglion cell projections (Soares and Mason, 2015), spine formation in the hippocampus (Awad et al., 2018), and perturbations of the circadian clock (Noda et al., 2019). Furthermore, electroporation as a technique to introduce DNA has been used in species from chickens (Muramatsu et al., 1997) to ferrets (Borrell, 2010). However, for our studies, we opted to validate our system using phenotypes previously shown to cause cortical radial migration defects in mice. The reasoning for this is as follows. First, mouse cortical IUE is one of the most heavily studied areas of neuronal development. Second, we felt that if Double UP was going to have problems replicating previous findings, it would be in early phenotypes because Cre would need to: be transcribed, be translated, locate Double UP, recombine and then for the overexpression/shRNA yield a result. We reasoned that of established phenotypes, migration deficits would be among those requiring the shortest amount of time for manipulation to have the desired effect.

It is important to emphasize that this manuscript has focused on migration. While no apparent differences were present in cell morphology, this was not examined closely and other metrics such as axon elongation or targeting were not tested. It will be important that anyone using Double UP confirms that for the metric being examined, green and red neurons behave similarly. While it has likewise not been tested, Double UP should work equally well in establishing internal controls for organoids, which are notoriously variable from one organoid to another.

By introducing an internal control and testing the validity of that control in several ways, we believe we have developed an easier to use, more rigorous version of IUE. Dependence on section matching is greatly reduced and variations in surgical technique will matter far less. Furthermore, other experimental paradigms are now much more feasible. For example, IUE can be performed on embryos from heterozygous-by-heterozygous crosses of mice because each embryo, whether it be wild type, heterozygous or homozygous, operates as its own control, no longer requiring well-matched electroporations from a matching genotype. Additionally, by introducing a tailored method of automated analysis with the TRON program, we have further reduced variability and bias in analysis. Implementation of Double UP and the TRON program allows the use of more rigorous statistical tests, which increases statistical power. Together, using Double UP and the TRON program allows for much faster collection of more reliable data.

*In utero* electroporation is a powerful and widely used technique, but is traditionally dependent upon extremely accurate and insufficient section matching. Brains have strong developmental gradients acting along multiple axes, increasing the difficulty of accurate section matching with regards to traditional IUE. By employing Double UP, experiments can now be performed within a single embryo, from a single surgery. Animal to animal variation is no longer an issue, inconsistencies in surgical technique are made inconsequential, and section matching is greatly reduced. Thus, implementation of Double UP can provide more rigorous data, while simultaneously reducing the number of animals, reagents, and time to complete experiments.

## Methods

### Materials Availability

Double UP, Double UP Rac1-V12, and Double UP Rac1-N17 are available through Addgene (#125139, #125136, and #125137, respectively).

Though not utilized in this manuscript, we have generated additional versions of Double UP that have been validated and are also available at Addgene. These plasmids are: Double UP superfolderGFP-to-mScarlet (Addgene #120261), Double UP mClover3-to-mScarlet (Addgene #120262), Double UP Halotag-to-mScarlet (Addgene #125138), Double UP mScarlet-to-mNeon (Addgene #125139), and Double UP-FRT (Addgene #141111).

pCAG-Cre and pCAG-ERT2-Cre-ERT2 were gifts from Connie Cepko (Addgene #13375, #13777). pCAG-iCre was a gift from Wilson Wong (Addgene plasmid #89573). pSico PGK Puro was a gift from Tyler Jacks (Addgene plasmid #11586).

For the internal control to be valid, it is essential that both Double UP and Cre plasmids contain the same promoter, and so these Cre plasmids are highly recommended for use in combination with Double UP.

Further information and requests for resources and reagents should be directed to and will be fulfilled by the Lead Contact, ED (ewdent@wisc.edu).

### Cell Culture Studies

CAD Cell lines were purchased from Sigma–Aldrich (08100805). Cells were cultured in Dulbecco’s Modified Eagle Medium/Nutrient Mixture F-12 (1056018, ThermoFisher Scientific) with 8% heat-inactivated Fetal Bovine Serum (Wicell) and 1% penicillin-streptomycin (15140122, ThermoFisher Scientific) in a humidified incubator at 37°C and 5% CO_2_.

For leakiness experiments (Figures 3D,E), CAD cells were transfected using Lipofectamine 3000, following manufacturer instructions. This resulted in an estimated 60% transfection rate, based on fluorescence.

### Animal Models

All mouse procedures were approved by the University of Wisconsin Committee on Animal Care and were in accordance with NIH guidelines. Timed matings were performed, and pregnant dams were used at embryonic day 14.5 (E14.5). Day E0.5 is the morning of sperm plug visualization. IUE was performed at E14.5, with embryos perfused either 3 or 4 days later, as specified in the text. Gender of embryos was not recorded. Pregnant females were housed individually. Prior to becoming pregnant, females were housed with three to four other females.

### *In utero* Electroporation (IUE)

Plasmid DNA was mixed before injection. For most studies (Figures 1, 2A–C, 3A, 4C–F and Supplementary Figures S1–S3) either no Cre, or 15 ng/μL of either pCAG-Cre or pCAG-CreER was mixed with 2 μg/μL of Double UP, Double UP Rac1-V12, or Double UP Rac1-N17. For knockdown studies (Figures 5A–C), 15 ng/μL pCAG-iCre was mixed with 1 μg/μL Double UP and 2 μg/μL of either pSico RapGEF2 or pSico scrambled. Plasmid DNA was then combined with Fast Green FCF to a final concentration of 0.05% Fast Green FCF, and loaded into pulled capillary needles. The dam was anesthetized with isoflurane, and a laparotomy was performed, exposing the embryos. The embryos were gently pulled out of the abdominal cavity. The capillary needles were inserted into the lateral ventricles, and approximately 0.25–0.5 μL DNA/Fast Green FCF was injected using a PicoSpritzer II (Parker Instrumentation). Electrical current was passed across the head, in five pulses of 40 V each lasting 100 ms on and 900 ms off using a CUY21 Electroporator (Bex Co., Ltd.). After the last embryo was electroporated, the embryos were inserted back into the mother, and the laparotomy was sutured closed. Embryos were allowed to develop normally for 3 or 4 days, depending on experiment (E14.5+3 or E14.5+4). Surgeries were performed on embryos from at least two different pregnant females for each experiment.

### Tissue Collection

Embryos were again exposed via laparotomy on the mother after deep anesthesia with isoflurane. Embryos were removed from the uterus one by one, chest cavity was opened, a small incision was made in the right atrium, and a needle was inserted into the left ventricle. Through this needle, the animal was perfused with approximately 1 mL of sterile saline and 3 mL of 4% paraformaldehyde (PFA) using an Instech perfusion pump, at the rate of approximately 1.25 mL per minute. Following perfusion, heads were removed and left in PFA at 4°C overnight before dissection. After the last embryo was perfused, the dam was euthanized. Following dissection, embryos were screened for positive signal using the mScarlet signal, which is easily visible in an intact, dissected brain. All brains containing mScarlet signal were further processed.

### Fixed Tissue Sectioning

After 16 h in PFA at 4°C, heads were transferred to PBS and brains were dissected. Brains were placed in 3% low melt agarose for 10 min at 42°C, then moved into 6% low melt agarose, and allowed to set on ice. After the agarose hardened, the brains were sectioned on a Leica VT1000S vibratome at 100 μm in PBS. Sections were stored for less than 1 week in PBS+0.2% sodium azide before being stained with 4’,6-diamidino-2-phenylindole (DAPI) and imaged.

### Section Preparation

DAPI was diluted to a final concentration of 2.4 nM in 0.4% Triton/PBS, and left on sections for 1 h on a gently rotating platform at room temperature. After 1 h, the sections were washed three times in PBS before being mounted in Aqua-Poly Mount (Polysciences). Slides were allowed to dry for at least 1 h and then imaged within 2 days.

### Section Matching

When section matching was used (Figures 1C,D and Supplementary Figure S1), every 100 μm section was collected and stored sequentially. All sections were treated with DAPI and mounted. The first slice to contain an uninterrupted CC was identified, and set as slice 0. All sections containing fluorescent signal were imaged and analyzed. One brain contained fluorescent signal in sections located before the first slice to contain an uninterrupted CC, but these sections were not analyzed as there was no matching section in the other brains examined in this way.

### Imaging

Confocal imaging was performed on a Zeiss LSM 800. Unless otherwise noted, all images were acquired at 12 optical sections, each 1 μm apart. 2 × 2 Tiles were collected with a 20x/0.8NA Plan Apochromat objective, with 2x averaging, and presented as maximum projections. Figure 3A was acquired at 47 optical sections, each 0.24 μm apart, with a 63x/1.4NA Plan Apochromat oil objective, with 4x averaging, and presented as a maximum projection. Unless otherwise specified, gain/laser power were altered between each image set to optimize image quality. Tiles were stitched together using the stitching tool in Zen 2.3 (Zeiss), and resulting images were analyzed using the TRON Program software described elsewhere in this manuscript.

### Western Blot Analysis

For leakiness experiments (Figure 3E) cells were transfected at 60% confluency with 10 μg of Double UP and either no pCAG-Cre or 1 ng pCAG-Cre using Lipofectamine 3000 (Invitrogen) following the manufacturer’s protocol. 48 h after transfection, cells were washed once with cold PBS before being lyzed with 300 μL NP-40 Lysis Buffer (Invitrogen) with Complete Mini (Roche) at 48 h post-transfection. Lysate was spun at 21,000 *g* for 10 min, and supernatants were flash-frozen and stored at -80°C until use. Samples were thawed and loaded onto a 4–10% SDS-Page gel, then transferred to PVDF membrane (Millipore). Membranes were blocked in 5% milk in 0.1% TBS-T, incubated with primary antibody overnight at 4°C, and blotted with a HRP-conjugated secondary antibody for 1 h. Antibodies used were mouse anti-HA (1:1000, sc-57592, Santa-Cruz), Mouse anti-Tubulin (1:10,000, T9026, Sigma), and goat-anti-mouse HRP (1:10,000, 115-035-174, Jackson). Protein bands were visualized using Pierce ECL Western blotting substrate (Thermo Scientific).

### Quantification and Statistical Analysis

Data were tested for normality using the Kolmorgorov–Smirnov test of normality. If data were normal, then a two-tailed *t*-test was performed. If data were not normal, then a Kolmorgorov–Smirnov two-tailed *t*-test was performed. For data comparing multiple brains at the same time, two-way ANOVA was performed. For these two-way ANOVA, reported *p*-values refer to the *p*-value associated with variation due to the differences in color within slices. *P*-values associated with variation due to differences between slices were not reported. Complete data are available upon request. All statistical tests were performed in Prism 8 (Graph Pad).

### Data and Code Availability

The TRON program was designed to perform reproducible, high throughput, and largely automated calculation of cell body location and distance from one or more user defined regions of interest. It was specifically designed for progenitor cells and migrating neurons, or neurons that have recently completed migration. We anticipate it will work well for most cell types, excluding highly branched and differentiated neurons. Most steps are automated, manual steps will be indicated. The TRON program largely utilizes ImageJ/Fiji tools. In short, images are processed via unsharp mask, gaussian blur, thresholding, erosion, and watershedding to transform cells into reduced cell bodies. These cell bodies are then run through a modified 3D objects counter to determine which cells in a similar XY location in different slices are the same or distinct cells. The distance from each cell to a user defined ROI is then calculated. A downloadable program, complete instructions for use, and a sample Double UP image file can be found here: https://go.wisc.edu/tron. Full code and associated instructions can be found here: https://go.wisc.edu/troncode.

## Data Availability Statement

The datasets generated for this study are available on request to the corresponding author.

## Ethics Statement

The animal study was reviewed and approved by the University of Wisconsin Committee on Animal Care.

## Author Contributions

RT and ED conceived the project. RT, JC, LG, KT, and KR executed the experiments. RT and ED wrote the manuscripts with input from all authors. ED supervised all aspects of the work.

## Conflict of Interest

The authors declare that the research was conducted in the absence of any commercial or financial relationships that could be construed as a potential conflict of interest.
