# Supplementary material for: Double UP: A Dual Color, Internally Controlled Platform for in utero Knockdown or Overexpression
Source: Front Mol Neurosci. 2020 May 20;13:82. doi: 10.3389/fnmol.2020.00082 (PMC7251070; doi:10.3389/fnmol.2020.00082)
Supplement: Supplementary file 1 [file Data_Sheet_1.docx]

**
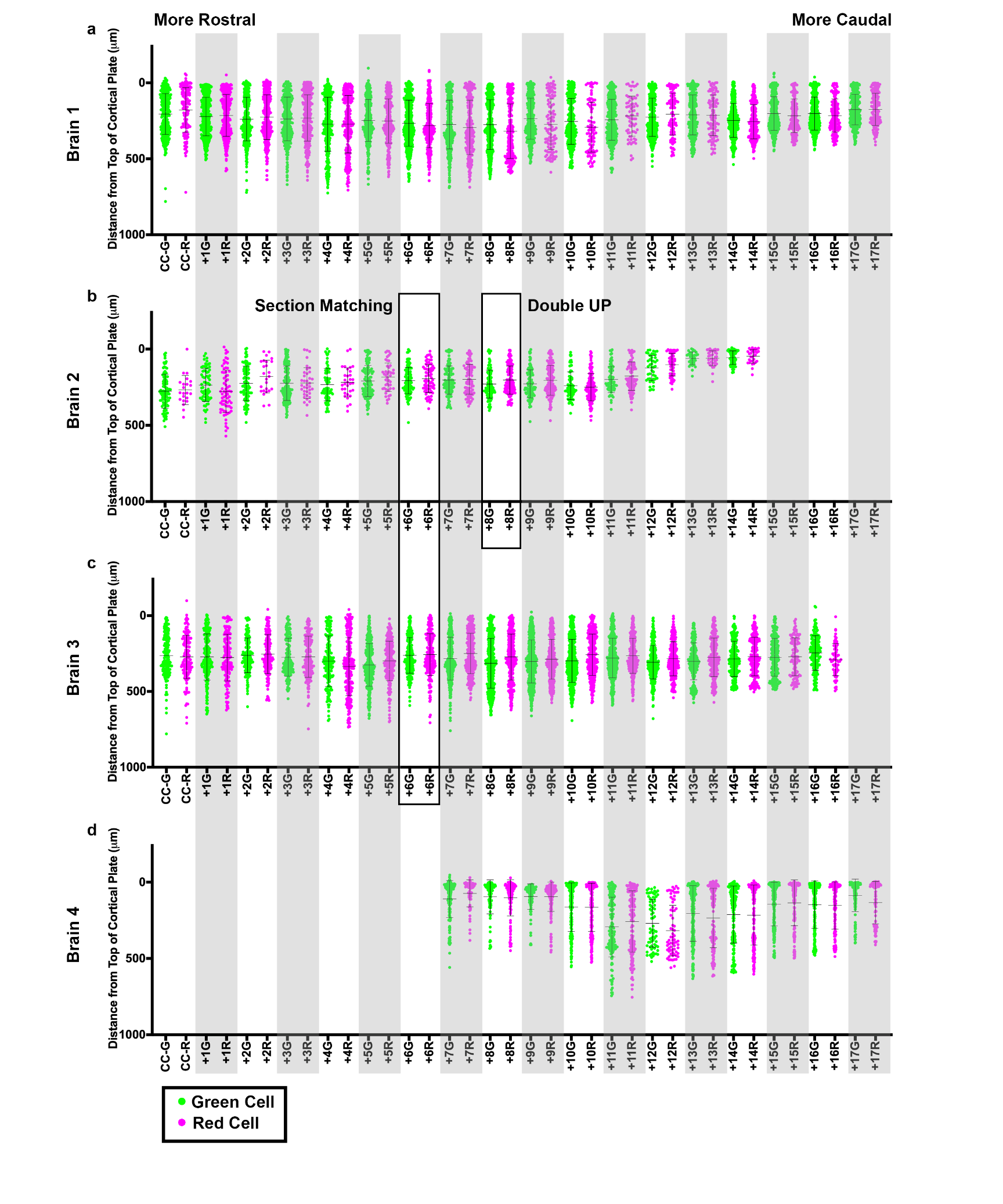
**

**Supplemental Figure 1: Full Data for Figures 1C and 1D.**

Comparison of migration data between and across brains. **(a-d)** Results for cell location of four different brains, all relative to the top of the cortical plate. Each dot corresponds to the location of a single cell, either mNeon (green) or mScarlet (red) relative to the top of the cortical plate. One example of section matching is shown between corresponding sections 12 in brains **b** and **c** (large boxed region). An example of the Double UP comparison is highlighted by the boxed region in section 8 of brain **b**. Lines within each population of red and green cells indicate mean and SD. Along the x-axis, G and R refer to all green (G) or all red (R) neurons within a section, while +1, +2, +3, etc. refer to how many sections caudal of the CC the section is located. Each section is 100µm in thickness. (n=118-1026 cells per section, approximately half green, half red)


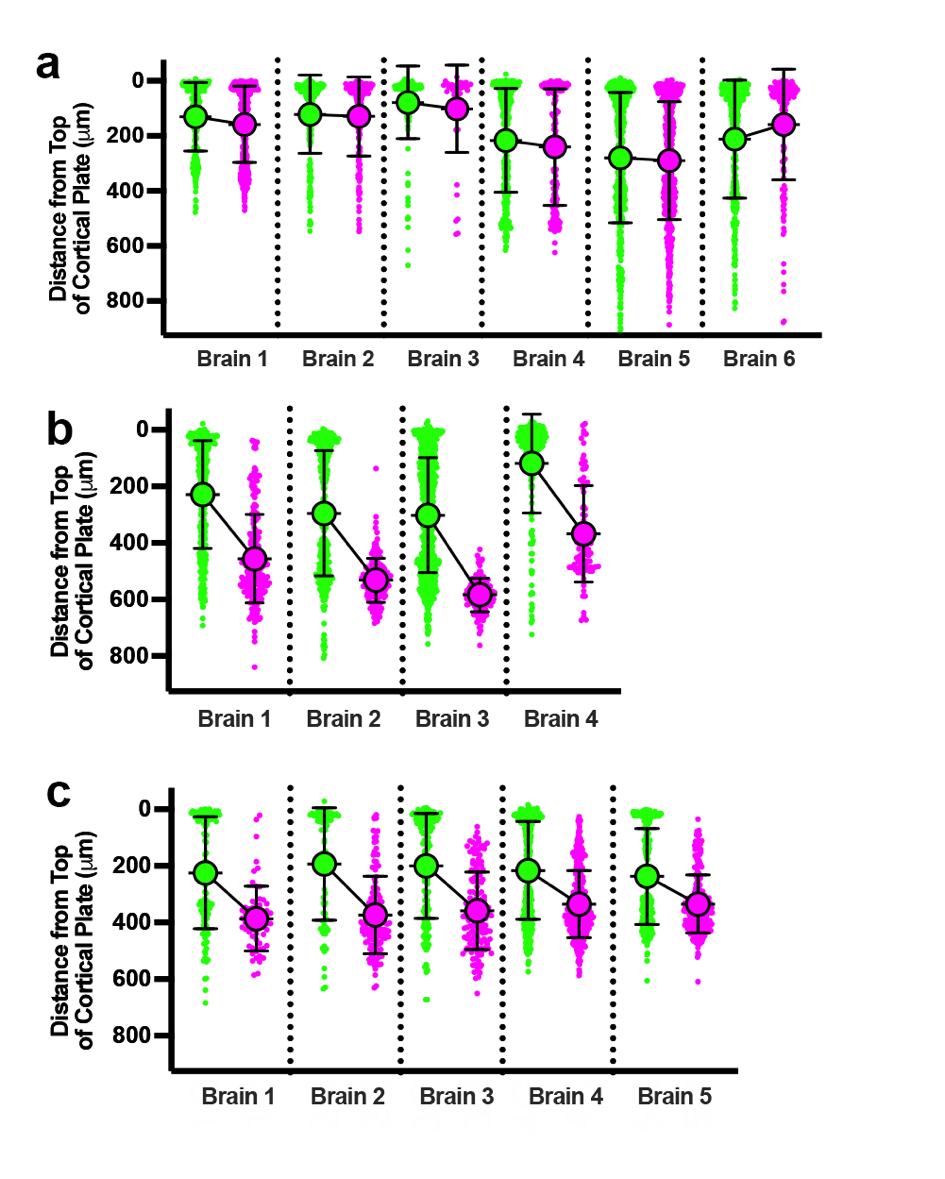


**Supplemental Figure 2: Full Data of Figure 4f.**

**(a-c)** Results for cell location for Double UP **(a)**, Double UP Rac1-V12 **(b)** and Double UP Rac1-N17 **(c)**, 6, 4 and 5 slices respectively, each from a different brain. Statistics were run only on the combined data presented in **Figure 4f**. Bars represent Mean and Standard Deviation.


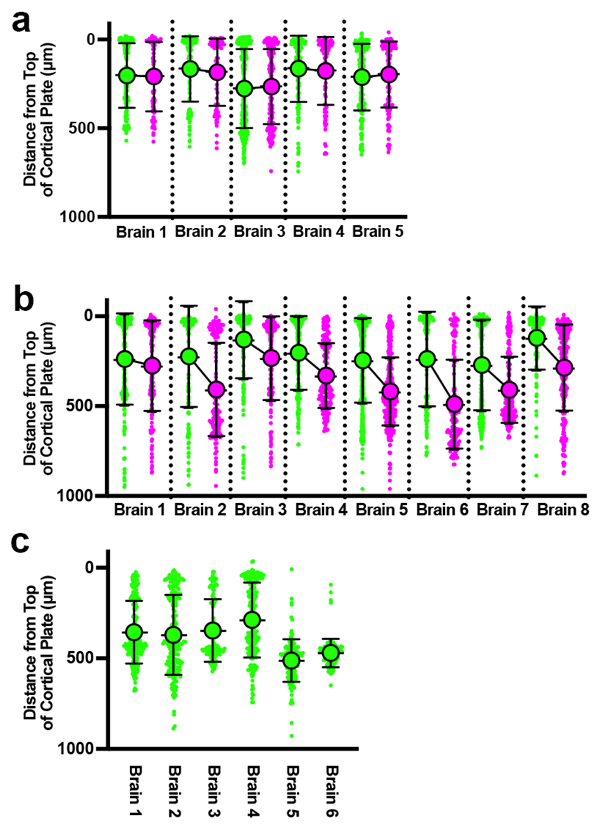


**Supplemental Figure 3: Full Data of Figure 5**

**(a-c)** Results for cell location for Double UP (1µg/µL) with pSico Scrambled (2µg/µL) and pCag-iCre (15ng/µL) **(a)**, pSico RapGEF2 (2µg/µL) and pCag-iCre (15ng/µL), **(b)** and pSuper RapGEF2 (2µg/µL) **(c),** 5, 8 and 6 slices respectively, each from a different brain. Statistics were run only on the combined data presented in **Figure 5c**. Bars represent Mean and Standard Deviation.
